# Factors Related to Preventive Behaviors against a Decline in Physical Fitness among Community-Dwelling Older Adults during the COVID-19 Pandemic: A Qualitative Study

**DOI:** 10.3390/ijerph19106008

**Published:** 2022-05-15

**Authors:** Yumi Kimura, Hiroshi Akasaka, Toshihito Takahashi, Saori Yasumoto, Kei Kamide, Kazunori Ikebe, Mai Kabayama, Ayaka Kasuga, Hiromi Rakugi, Yasuyuki Gondo

**Affiliations:** 1 Graduate School of Human Sciences, Osaka University, Suita 565-0871, Osaka, Japan; syasumoto@hus.osaka-u.ac.jp (S.Y.); ayaka826spring.sun@gmail.com (A.K.); y.gondo.hus@osaka-u.ac.jp (Y.G.); 2 Department of Geriatric and General Medicine, Graduate School of Medicine, Osaka University, Suita 565-0871, Osaka, Japan; akasaka@geriat.med.osaka-u.ac.jp (H.A.); rakugi@geriat.med.osaka-u.ac.jp (H.R.); 3 Department of Prosthodontics, Gerodontology, and Oral Rehabilitation, Graduate School of Dentistry, Osaka University, Suita 565-0871, Osaka, Japan; toshi-t@dent.osaka-u.ac.jp (T.T.); ikebe@dent.osaka-u.ac.jp (K.I.); 4 Department of Health Promotion System Science, Division of Health Science, Graduate School of Medicine, Osaka University, Suita 565-0871, Osaka, Japan; kamide@geriat.med.osaka-u.ac.jp (K.K.); kabayama@sahs.med.osaka-u.ac.jp (M.K.)

**Keywords:** preventive behaviors, older adults, physical fitness, frailty, sarcopenia

## Abstract

Older adults face the concern of developing frailty and sarcopenia due to an inactive lifestyle during the coronavirus disease 2019 (COVID-19) pandemic. This study aimed to reveal the preventive behaviors taken by older adults who perceived a decline in physical fitness during COVID-19 and analyze the background factors which promoted such behaviors using a qualitative study design in 2020. The participants were recruited through the cohort study of Japanese older adults who were aged 79–81 and had not been diagnosed with sarcopenia previously in 2019 and perceived their physical fitness to have declined during the pandemic. The interviews of 19 participants were analyzed using thematic analysis. The participants engaged in five types of preventive behaviors to counter declining physical fitness: “walking”, “exercising at home”, “improving daily diet”, “maintaining a daily routine”, and “taking a good rest”. Four themes were extracted pertaining to backgrounds of such preventive behaviors: “feeling anxiety and mental pressure”, “available networks with family and neighbors”, “prior experiences of behaviors”, and “access to information”. Anxiety due to lifestyle changes during the pandemic was the primary reason for the behaviors. This study can be a useful guide for undertaking possible measures to prevent frailty during future pandemics.

## 1. Introduction

Given population aging and the rising care burden, most aged societies in the world urge measures to prevent frailty and sarcopenia as a public health policy to delay the onset of disability [1]. The benefits of physical exercise for preventing or improving frailty in older adults have been previously studied, including in observational and intervention studies [2]. To address sarcopenia (physical frailty), guidelines suggest that nutrition intervention and exercise are effective [3,4]. However, it is still unclear what encourages older adults to engage in preventive actions, such as engaging in exercise or improving diet, especially under specific circumstances, such as restrictions due to the pandemic.

Since the coronavirus disease 2019 (COVID-19) pandemic was officially declared a public health crisis worldwide by the World Health Organization (WHO, Geneva, Switzerland) in March 2020, vulnerable populations, including older adults, have been affected directly and indirectly [5,6]. Japan, a super-aged society, with an aging rate of 27% and more than 18 million people aged 75 and older, has experienced a severe social impact due to the pandemic. The Japanese government declared a state of emergency and asked the public to avoid leaving their residences to prevent infection; thus, people were discouraged from engaging in community activities [7,8]. Older adults face a higher risk of infection from COVID-19, and are more susceptible to impaired physical and mental functioning due to prolonged avoidance of activities and restricted travels [9,10]. Yamada and colleagues conducted an online survey and found that physical activities of older adults aged 65–84 years and living in urban areas decreased by 65 min per week [11]. Later, they reported that older adults who lived alone and were socially inactive were less likely to improve their physical activity levels after the first wave of the pandemic [12,13]. Older adults with frailty have also experienced decreased physical activity, lower extremity muscle strength, and diversity in food intake and quantity [10], leading to an increased risk of sarcopenia. Thus, the negative impact of COVID-19 on older adults is becoming more apparent through quantitative studies.

In a recent study conducted by Makizako et al., a survey of 774 community-dwelling Japanese older adults revealed that 43.8% of participants experienced a decline in physical fitness during the state of emergency of COVID-19 [14]. The study also reported that the percentage of older adults who perceived a decline in physical fitness had fewer exercising habits during the period. Hence, exercise habits might have a significant effect on declining physical fitness. An online survey by Makizako et al. also reported that among adults aged 40–69 years with a perceived decline in physical fitness, physical activities had decreased during April 2020, and were not resumed even in October 2020 when the restrictions were lifted [15]. This suggests that the exercise habits may not readily recover once lost, even after the pandemic ends, which might have serious consequences for older adults.

Although the quantitative surveys showed a decrease in physical activities among older adults during the pandemic, there is a lack of understanding about the actual situation of the daily life of older adults. Because the pandemic brought about a new social situation which no one had ever experienced before, the lifestyle changes of older adults should be explored in various methods. Thus, qualitative study approaches are increasingly important to understand how older adults experienced the pandemic and tried to promote their health in response to the lifestyle changes. There are not many previous studies using qualitative methods during the COVID-19 pandemic because of the risks of infection through face-to-face interviews. Some previous studies were challenged with describing the lifestyle changes of Japanese older adults during the pandemic; a qualitative study reported older adults’ perception of the effects of the stay-home restrictions on their daily life [16], and another described the difficulties experienced along with lifestyle changes among day-care users [17]. However, the positive attempts among older adults to maintain their health, such as exercise or other preventive behaviors conducted during the restriction period, are still unknown.

It is important to explore various preventive behaviors of older adults using a qualitative approach to understand which behaviors or actions were performed during the period of perceived decline in physical fitness. Moreover, the reasons and backgrounds for performing such actions might be critical for suggesting possible strategies to support preventive actions. Thus, this study aimed to understand what kind of preventive behaviors were taken by older adults who perceived a decline in physical fitness during COVID-19 and explore the background factors that triggered and motivated such behaviors using a qualitative approach.

## 2. Materials and Methods

### 2.1. Study Design

This study was conducted as a part of the longitudinal cohort study, the SONIC (Septuagenarians, Octogenarians, Nonagenarians Investigation with Centenarians) study, which is a cohort study of community-dwelling older adults that began in 2010. We adopted a qualitative research design to explore the actual behaviors and their backgrounds among older adults during the pandemic, using semi-structured interviews following the Standards for Reporting Qualitative Research (SRQR) [18]. This study was performed in compliance with the Declaration of Helsinki and was approved by the Research Ethics Committee of the Graduate School of Human Sciences, Osaka University (approval number: OUKS1918).

### 2.2. Participants

The participants of this study were recruited from the SONIC study [19]. The study participants were aged 79–81 in 2020 and living in Itami and Asago in Hyogo Prefecture, Japan. Itami is representative of typical cities in the urban area, whereas Asago is a farming town representative of rural towns located in mountainous areas in the prefecture.

A questionnaire survey was sent through the post to the participants in August 2020 to determine the physical and mental health of older adults during the state of emergency imposed by COVID-19 (April–May 2020). A total of 269 participants living in Itami and Asago responded to the appropriate questionnaires of this study (response rate: 54.2%). [20]. Among them, the participants who had not been diagnosed with “major sarcopenia” in the previous survey of 2019 (*n* = 252) were included in the study. Sarcopenia status was assessed during the health-check survey in 2019 according to the guidelines of the Asian Working Group for Sarcopenia (AWGS), and categorized as minor and major sarcopenia prior to this study to exclude the participants who had already diagnosed with major sarcopenia before the pandemic period [21]. Among them, 94 participants perceived declined physical fitness and were randomly selected for a telephonic interview. In total, 21 participants were interviewed; however, 2 were excluded from the analysis because 1 was hospitalized during COVID-19 and did not reflect the situation we were trying to study, and the other interview was not lengthy enough, since the conversation ended within two minutes. Finally, a sample of 19 participants (7 men and 12 women) was analyzed.

### 2.3. Data Collection

Before the interview, the demographic information (including age and sex) and contact details of the participants were obtained from the questionnaire survey in 2020. We could not conduct the health check to assess sarcopenia status in 2020 because of the pandemic. Thus, the information about the self-rated changes in body weight and decreased physical fitness during the pandemic were evaluated by the questionnaire, as described above.

From September to December 2020, the authors conducted semi-structured telephonic interviews with the participants. The interview guide included open-ended questions on the preventive actions taken regarding perceived decline in physical fitness and the reasons for performing the preventive actions. The interviewer called the participants from the university room to ensure privacy. The purpose of this study was explained, and informed consent was obtained before the interview. The participants were interviewed by three researchers, and the time period varied between 6 and 24 min. All conversations were recorded and transcribed for analysis.

### 2.4. Data Analysis

The verbatim transcripts of the 19 participants were analyzed inductively. A qualitative descriptive analysis was conducted, which is widely used in nursing research [22]. The following steps were taken: (1) the recorded data were transcribed verbatim, (2) the data were delimited according to their characteristics about what and how the behavior was conducted, and codes were assigned that reflected the content of the delimited data, and (3) these codes were repeatedly examined to account for similarities and differences in the recorded data and were organized and integrated into themes. We examined the similarity and originality of the content of the semantic codes by collecting several similar codes. In the process of analysis, the three researchers carefully reviewed and confirmed the classification to ensure intercoder reliability [23]. MAXQDA Analytics Pro (2020 ver.) was used to import, organize, and analyze data.

## 3. Results

The characteristics of the participants are listed in Table 1. Data on the changes in body weight before and after the state of emergency were obtained from the questionnaire. BMI and sarcopenia status, as assessed by the survey in 2019, were also obtained from the SONIC study. The significant actions taken to deal with declining physical fitness are listed in Table 1. Each participant explicitly discussed their activities and behaviors during the restriction period due to COVID-19, which are summarized in Table 2 by categorizing the types of behaviors. Five participants shared that they “*did not or could not do anything*” for their declining physical fitness, despite being aware of their physical changes.

We identified five types of preventive behaviors undertaken by participants for declining physical fitness: “walking”, “exercising at home”, “improving daily diet”, “maintaining a daily routine”, and “taking a good rest”, as listed in Table 2. The narratives pertaining to “walking” varied, including negative views such as “I am no more “*genki* (energetic)” so *I just tried to take a short walk around my house*;” and high-intensity walking such as “*I walk for an hour every day*”, “*I always ensure more than 5000 steps with step counter*”. The category of “exercising at home” included exercising with television (TV) programs, weight training with machines or squats, simple stretching, yoga, and so on. The participants who answered that they improved their diet ate more protein, had three meals of a sufficient quantity, and ate specific foods recognized as healthy, such as soy milk and garlic. As listed in Table 2, the narratives of those who “could not do anything special” included positive behaviors such as trying to maintain daily tasks such as gardening, cleaning home, or a passive attitude towards resting, such as taking a nap. The participants living in rural areas talked about their daily routines in the neighborhood environments, such as “going to fields everyday” or “continue farming and gardening” more often than the ones living in cities. One participant living in the city said, “*Exercising was never my habit since I was young. (During COVID-19), I tried to maintain daily routines without becoming too tired*”. He started to read newspapers and wrote down some *Kanji* (*Chinese character*) words from the article as a daily routine to maintain a good rhythm and maintain his mental health condition. Another participant stated that “taking a good rest”, specifically taking a nap, as part of daily routine helped her prevent weakened physical strength.

### 3.1. Reasons for Preventive Behavior

We extracted four themes representing the broad categories of reasons or backgrounds that enabled older adults to take preventive actions or behaviors when they realized that their physical fitness was declining. These included: “feeling anxiety and mental pressure”, “available networks with family and neighbors”, “prior experiences of behaviors,” and “access to information about behavior”. These themes include several subthemes, as shown in Table 3.

#### 3.1.1. Feeling Anxiety and Mental Pressure

Every participant spoke of anxiety when talking about declining physical fitness. For example, one participant expressed “realizing weakness gradually” during daily tasks such as cleaning rooms. Another participant realized a considerable physical deterioration after his bypass surgery (performed for a problem with coronary arteries), which triggered his realization of the importance of health, especially during the pandemic, and he started high-intensity exercise at home using a bicycle machine.

*“I do feel that my physical strength is gradually weakening. Just vacuuming, cleaning, and other such things have become a huge deal, making me so exhausted. I was not like that before COVID. I have reached this age, so I have to do something to prevent my physical strength from deteriorating even more”.*

*“When I did (the surgery), my legs and lower back, especially the muscles in my legs, became so weak. Hence, I bought quite a new health equipment; a bicycle pedal, a hanging apparatus, and such. I really put effort into it at home, as I cannot go out and am getting weaker without doing anything”.*

Apart from being weak and declining physical fitness, a feeling that enhances anxiety is frailty and dependence in the future. A woman expressed her feelings of anxiety and tried to move around to make herself feel better. Another participant spoke of fear of the future in the context of being a burden to her children.

*“It will be very troublesome if my body stops functioning. It (the body) moves quite a bit if I do even slight exercises daily. …I tell myself. I do not want to become a burden, so I have to care for myself for my own good. The fear is when you cannot function anymore”.*

*“I think what is most important is not to be a burden to the younger people. I live alone, and who knows what kind of illness I might get, so I hold this feeling of not being a burden to my children”.*

#### 3.1.2. Available Networks with Family and Neighbors

Some participants began to walk or exercise because of the influence or reactions of the people around them. They had good relationships with family members or friends, who motivated them or asked them to walk together.

*“My children kept asking me, “mom, do something; otherwise you will be senile” so I came to feel like, oh, I must do something”.*

*“My friend reached out to me suggesting “let’s go for a walk” and so I went out for a walk with her, and then it has become a routine now”.*

The social networks of the older adults in the community, such as being a part of a senior citizens’ association, positively affected their motivation to take action, even when the club activities were not actually operational during the COVID-19 restriction period.

*“There (the senior citizens’ club), you get to interact with people and be social, so I used to join for a long time. Now I do not go to the club, so I can at least exercise at home. I learned several ways of simple exercises, so I do that at home”.*

#### 3.1.3. Prior Experiences of Behaviors

All participants who spoke of being engaged in physical activities had experience of the behavior before the COVID-19 pandemic. Some said that they were doing exercise at the senior citizens’ club regularly before the pandemic, and others said that they learned yoga more than 20 years ago, and this pandemic was a trigger that motivated them to participate once more in yoga practice. They could conduct the activities because they had priorly engaged in them, which made them familiar with certain exercises and helped them engage in or restart these behaviors during the pandemic, when they realized their physical fitness had declined.

*“I now do a little yoga in the house. The community center is closed right now, so I remembered yoga, I used to learn before… in my fifties for about five years. I sometimes do some poses that are easy to move. I have some knowledge of how to move my body, so sometimes I do what I like”.*

The participants who had a habit of going to the gym before the pandemic were likely to exercise or engage in easy stretches at home, since they were aware of their merits. Moreover, physical activities and daily routines, such as gardening or farming and rest, were also recognized as essential behaviors or attitudes in preventing feeling fatigued and maintaining physical strength, which was realized because of their experiences before the pandemic.

*“If I’m moving around a lot, I get really tired at night and feel that I am losing my physical strength, so I try to rest during the day. I try to take a nap; If I rest, I know I can get back on my feet a little”.*

*“I go to the field (she is a farmer) every day, nothing out of the ordinary. However, I think that for now (during the pandemic), what is important is to continue. It does not have to be a workout. If you move, the body moves. At least I believe so”.*

#### 3.1.4. Access to Information about Behaviors

Obtaining information during the pandemic was crucial for the participants to change their lifestyle or begin exercising. Television was the primary means for the older adults to obtain information, followed by radio, newspapers, books, or magazines. Those who could access adequate information could start the preventive behavior according to the information. Additionally, they could try to incorporate new activities or healthy eating advice into their daily life according to their preference or feasibility and decide if it yielded fruitful results.

*“You can’t really go outside, so I was contemplating what to do as TV often shows different types of exercises. I just happened to read in the newspaper that squats are good. Therefore, I started with squats 100 times a day before bed. The weakening of my legs and lower back is prominent, so it’s to help that”.*

*“I feel more “genki” (energized) when I am consuming meat, and that’s also often on TV. It says that older people should eat (meat), however many grams a day…, I cannot remember well, but the less amount is not good or something like that. Since I live alone, I try to eat more meat”.*

*“I have done the radio exercise but, considering my lower back and knee pains, it’s not very effective. I rather followed the books that the doctors wrote, if you continuously do those exercises, it does help”.*

## 4. Discussion

This study aimed to explore the backgrounds and reasons which enabled older adults to take action when they became aware of their declining physical strength due to COVID-19 restrictions. A variety of preventive behaviors taken by the participants were confirmed through interviews. Compared to the quantitative research studies, which determine the levels of activities undertaken, our study employed qualitative interviews to highlight the actual situation and differing range of health-promoting activities in community-dwelling older adults. From the interviews of 19 healthy participants that had not been diagnosed with major sarcopenia by the previous survey, some said that they realized they had become physically weaker after the pandemic. Although the authors tried to find the difference in behavioral characteristics between those with minor sarcopenia and without (robust status) before the pandemic, no typical characteristics were found that were associated with the behaviors shown in Table 1. Contrary to the previous study by Makizako and colleagues, which pointed out that older adults who perceived a decline in physical fitness had fewer exercising habits during the pandemic [14], our study showed the narratives of the participants, which illustrate various types of preventive behaviors, including exercising performed to counter the decline in physical fitness.

This study focused on the background factors that can help prevent a decline in physical fitness; however, the narratives of older adults who could not perform any positive activities for maintaining physical fitness are also worth discussing.

*“I was conscious, but I could not get myself to do anything. I mean, given my age. I have not been (to the sports club since COVID-19′s restraints) in a while, and now, really, the motivation just does not come to me”.*

*“In the sense, since I am not going out anymore, I have lost interest in what I wear. Hence, I just wear old clothes; I cannot dress up and do make up anymore”.*

These narratives reflect lowered motivation, a worsening mental status, and feelings of surrender in older adults because of their age and were told more by those who reported losing weight during the pandemic. Participants’ previous body weight and sarcopenia status in 2019 did not seem to impact their ability to improve their physical fitness during the pandemic in 2020. A previous study reported that the prevalence of depressive symptoms among community-dwelling older adults in Japan increased slightly during the COVID-19 restriction period [9]. Thus, interventions for older adults, primarily focusing on promoting mental health and preventing frailty and sarcopenia, must be realized, especially during the pandemic.

Fatigue is a subjective sense of tiredness and a lack of energy, and it is one of the core items of the frailty concept [24]. During the interview, most older adults discussed their “declining physical fitness” while implying almost the same meaning as “fatigue” or “weakness”. This may be because the Japanese word for physical fitness is “*tairyoku*”, meaning “power of the body”, written in *Kanji* (Chinese character). Oppositely, they often used the word “*genki (energized)*”, when talking about recovering from the state of fatigue. Hence, the narratives of declining physical fitness are perceived in the context of fatigue and a loss of energy. A previous study conducted in Spain reported that self-reported fatigue during lockdown due to COVID-19 had an inverse association with physical activity levels [25]. This study obtained narratives about preventive actions taken in response to perceived fatigue (feeling weak), such as “maintaining a daily routine” and “taking a good rest”, which seemed to be passive behaviors compared to exercise or dietary changes. However, these behaviors are reasonable and positive enough for the management of physical decline, with due consideration to their age, as the participants were around 80 years old. In the context of these narratives, we could deduce that “fatigue” (used by them) implied physical and mental fatigue. Mansfield et al., in a study on older adults, reported that self-reported deteriorating health, such as pain and depressed mood, can act as barriers to the ability to exercise [26]. At the same time, they suggested that these barriers could also act as motivators.

Being aware of a decline in health might motivate older adults to start activities, as anxiety and pressure about their physical health made them feel that “*I have to do something*”. Thus, risk perception could be crucial for the undertaking of certain behaviors, especially during the pandemic situation, which is unusual. The health belief model is a traditional model describing how people engage in health-promoting behavior [27]. Risk perceptions can accelerate preventive behaviors [28,29]. Schutzer and Graves reviewed age-specific barriers and motivations to exercise in older adults, and suggested that motivators and barriers are often intertwined, making it difficult to isolate factors specific to the cohort [30]. This study specifically focused on behaviors in response to the heightened perception of risk during pandemics and extraordinal situations. Here, anxiety and mental pressure could be motivators, but were also barriers that often led to depressive moods in older adults during the pandemic.

The narratives of mental pressure were also present in relation to other people, such as “*my children told me to do something*”. This also increased their awareness about health behaviors, and made us consider the importance of available networks with family or neighbors. In line with a previous study by Yamada et al., which reported that older adults who lived alone were less likely to improve their physical activity levels during the pandemic [12], our study could suggest that the presence of someone to motivate them has a significant impact on commencing health behaviors. Social connections are reported to be essential factors in enhancing physical activity [31]. The evidence surrounding the relationship between social support and physical activities in older adults has been accumulated in prior research. A study suggests that social support, especially that of family members, motivates older adults to engage in more physical activities, and emotional and informational support were associated with higher intentions [32]. Although the older adults living alone were less active than those who lived with their children, Pérez et al. reported that the pre-lockdown situation of social contacts with friends or neighbors was related to higher levels of physical activity and contributed to maintaining or improving physical activities among Spanish older adults between pre-lockdown and lockdown [25]. Thus, connections to friends or neighbors, which may differ from those with family members, can also be crucial in motivating them to engage in social activities. In addition, the impacts of communication with family members and friends on health behaviors were more often noted by female participants than males. A case study on the community-based health program in Japan reported different attitudes toward participation among male and female participants, especially regarding communication with others [33]. The gender differences should be taken into account when considering the available networks of family and neighbors as a background factor for health promotion.

Our study also found interesting connections between health behavior and past experiences. Concerning physical activities, all the participants who could engage in physical activities or exercises during lockdown had priorly engaged in similar behavior before the pandemic. Regarding the impact of past exercise habits, few studies have been performed among the older adult population, and some evidence suggests that exercise patterns in childhood can adversely affect levels of exercise in adulthood [34]. From participants’ narratives, such as, “*If I keep moving, my body will function*” and “*if you continuously do those (exercises), it does help*”, it emerges that they believe doing a minimal amount of exercise is better than no exercise. This type of belief regarding health behavior, such as self-efficacy, has been long studied by the behavioral science academic field.

The concept of self-efficacy has consistently been identified as an essential determinant of exercise behavior in various populations and many types of behavioral learning throughout the scientific literature. Defined as an individual’s belief in their ability to successfully perform a specific behavior, self-efficacy plays a central role in Bandura’s social cognitive theory [35]. Regarding the change in the exercise behavior stage, self-efficacy is a product of expectations (perceived ability to achieve a specific behavior) and outcomes (expected success the behavior will provide); thus, self-efficacy exerts a consistently powerful influence on the exercise behavior of older adults [36,37]. A review of the physical activities of older adults suggested that psychological factors such as motivation and self-efficacy are linked to higher levels of physical activity in older adults [38]. In addition to physical activities, some participants also reported that good rest or sleep, and maintaining a daily routine, such as farming, were also consciously conducted during the pandemic, because they believed it to be helpful due to their past experiences. These lifestyle behaviors might have been influenced by environmental factors. The older adults living in rural areas had been going to the fields and/or engaging in gardening for many years, and were led to believe that it is good to continue these activities, which might have motivated them during the pandemic situation. However, responding to a rapid change caused by the pandemic requires a different approach than the available models of health behavior, such as self-efficacy or health belief models, which are based on long-term transformation stages, and might not be simply applicable in such emergencies. Hence, our study sheds light on the actual voices of older adults who tried to cope with their physical decline during the pandemic restrictions.

In addition to their own experiences, the new information relating to health promotion during the COVID-19 pandemic was crucial to motivating participants to start certain types of exercise or improve their diet. During the pandemic, access to adequate information was often a significant challenge, especially for older adults. In particular, using digital devices is often difficult for older populations. Digital inequalities, which are already recognized as a determinant of health, have become more severe in the pandemic era [39], leading to social and digital exclusion [40]. A previous survey of community-dwelling older adults aged 75 years and above in a town in Japan during COVID-19 found that the use of information and communication technology was associated with voluntary exercise [41]. This suggests that information access and connections with others through digital devices can motivate older adults to start activities. This study’s participants did not mention digital devices, such as smartphones (except one who mentioned using a step counter when walking), but they admitted they often start something new after referring to TV, the radio, newspapers, and sometimes magazines. However, a variety of information seemed to confuse them as per their narratives. Those who performed activities could select the adequate information to choose adequate activities for themselves, which needed a certain level of health literacy. Those who could access this information may have already had high motivation to maintain or promote their health. A previous review paper identifying the motivators and barriers to physical activities reported that intrapersonal factors such as knowledge and attitudes of individuals had more impact than interpersonal and environmental domains [42]. Thus, access to information can act as a trigger, and also indicate the capacities of older adults regarding health literacy and consciousness.

Finally, the novelty of this study is that it revealed the difference in the backgrounds of health behaviors between a typical situation and a pandemic. Our qualitative study suggests that increased anxiety in a short period and sudden lifestyle changes (including restrictions on going out and socializing) makes older adults take preventive actions when they perceive declining physical fitness. Although the authors discussed four themes that acted as the backgrounds of the participants who performed preventive behaviors, it is notable that these four themes interacted with each other; individual participants talked about complex backgrounds and reasons across the themes.

This study had some limitations. First, the interview was conducted telephonically to avoid the risk of infection; thus, they could not capture non-verbal cues during the conversation. Second, due to the difficulty of the interview during the pandemic, it was challenging to reach the community-dwelling older adults (79–81 years old) via telephone calls; thus, the sample size was relatively small, although we carefully designed the study following the standard requirements for the qualitative methodology. In addition, we did not evaluate the actual effects of those preventive behaviors. Whether they actually worked effectively for preventing sarcopenia or frailty was not our focus, even though some participants noted that they realized the effects on self-perceived physical weakness or fatigue. This study employed an exploratory qualitative research method rather than a definitive and hypothesis-testing method. Thus, the actual situations of the older adults who tried to cope with their decline in physical fitness during the pandemic were revealed, and the backgrounds of their behaviors were analyzed. Further studies are needed to observe the behavioral changes for a longer period, and to evaluate their effects.

## 5. Conclusions

The study revealed various kinds of preventive behaviors undertaken by older adults when they perceived a physical weakness during the COVID-19 pandemic. Anxiety and mental pressures due to lifestyle changes during the pandemic were primary reasons and triggers for the behaviors, and past exercise habits, available support from relationships, and access to information were factors related to older adults’ performing of those actions. The qualitative approach enabled us to present the actual experiences of older adults during the pandemic situation. This study might help healthcare professionals in devising strategies for preventing frailty or sarcopenia to provide interventions and possible measures for older adults during future pandemics.

## Figures and Tables

**Table 1:** Baseline characteristics of the participants.

Case	Gender	Major Actions Taken to Deal with Declining Physical Fitness during the COVID-19 Pandemic	Self-Rated Changes in Body Weight during COVID-19 (in 2020)	BMI in 2019	Sarcopenia Status in 2019
A	Female	Walking in a park for an hour	None	16.9	Minor
B	Male	Exercising at home using a bicycle machine	Loss	20.9	Minor
C	Female	Nothing	Loss	21.6	Minor
D	Female	Exercising and squats at home everyday	None	23.9	Minor
E	Male	Exercising at home	Loss	19.3	Minor
F	Female	Taking a small walk, yoga at home	Loss	17.8	Minor
G	Female	Not able to do anything	Loss	26.5	None
H	Male	Walking around the house	None	22.6	None
I	Male	Nothing	Gain	21.4	None
J	Female	Walking (counting steps)	Gain	24.6	Minor
K	Male	Nothing	Loss	18.5	Minor
L	Female	Exercising at home, eating healthy foods	None	30.0	None
M	Male	Nothing	Gain	23.3	None
N	Female	Walking	Gain	23.3	None
O	Female	Taking a walk around house	Loss	27.2	None
P	Male	Squatting	Gain	29.0	Minor
Q	Female	Waking for an hour, yoga at home	None	20.1	None
R	Female	Nothing in particular (tried to maintain daily activities, such as farming and gardening)	None	24.3	None
S	Female	Nothing in particular (tried to get more rest and take naps)	None	22.1	Minor

**Table 2:** Types of behaviors engaged in when declining physical fitness was perceived during the COVID-19 pandemic.

Types of Behavior	Variables	Cases
Walking	Passive walking (counting steps, measuring time, etc.)	7
	Taking a walk (around the house, in the neighborhood, etc.)	
Exercising at home	General exercising	8
	Squats, weight training	
	Yoga, stretching	
Improving daily diet	Eating high-protein diet	6
	Eating three meals per day even if not hungry	
	Eating certain foods recognized as healthy	
Maintain daily routine	Tried to maintain a daily living	3
	Tried to continue gardening or home farming	
Taking a good rest	Did nothing special but rested	2
	Intended to take naps during the day	

**Table 3:** Reasons and triggers for the preventive behaviors against declining physical fitness during the COVID-19 pandemic.

Themes	Subthemes
Feeling anxiety and mental pressure	Awareness of the current situation of being weak
	Fear of being a burden to children
	Anxiety of being frail and immobile in the future
Available networks with family and neighbors	Relationships with family members who provide support
	Friends’ or neighbors’ invitation to go out
	Belonging to certain groups
Having prior experiences of behaviors	A habit of going to the gym before the pandemic
	Experience with yoga or sports in the past
	Realizing merits of daily behaviors
Access to information about behavior	Having health literacy and consciousness
	Reading books or magazines, newspapers, watching TV

## Data Availability

The datasets of the current study are available from the corresponding author on reasonable request.
